# Rationale, design, and analysis of combined Brazilian household budget survey and food intake individual data

**DOI:** 10.1186/1471-2458-8-89

**Published:** 2008-03-17

**Authors:** R Sichieri, RA Pereira, A Martins, ABPA Vasconcellos, A Trichopoulou

**Affiliations:** 1 Social Medicine Department, State University of Rio de Janeiro, Rio de Janeiro, Brazil; 2 Department of Nutrition, Federal University of Rio de Janeiro, Rio de Janeiro, Brazil; 3 Brazilian Office of Geography and Statistics, Rio de Janeiro, Brazil; 4 Department of Food and Nutrition Policy, Brazilian Ministry of Health, Brasilia, Brazil; 5 Department of Hygiene and Epidemiology, University of Athens Medical School, Athens, Greece

## Abstract

**Background:**

Data on food intake at the individual level and its statistical distribution in population groups defined by age, gender, or geographic areas are important in planning public health and nutrition programs. However, individual-based surveys in representative population samples are expensive to perform.

**Methods/Design:**

In Brazil, an individual based survey is under consideration to be conducted alongside the household budget survey (HBS), which will be carried out in 2008–2009. This paper presents the methodological framework of dietary data collection and indicates the directions to combining both sources of data.

The 2008–2009 Brazilian HBS sample will include 60,000 households. Of the selected HBS households, 30% will be randomly sampled to gather data on individual food intake. Therefore, individual dietary intake data is expected to be gathered for 70,000 individuals. Data collection procedures will comprise: completion of a diary with information regarding food purchases during a seven-day period; registration of all items consumed during two non-consecutive days for all 10 year-old or older members of the household. The sample will be large enough to capture the variation between individuals, and the two records will assure the estimation of the variation within individuals for food groups, energy and nutrients. Data on individual dietary intake and food family budget will be stratified by the five regions of the country and by rural or urban. A pilot study has been conducted in two states, and it indicated that combining individual and budgetary data in a survey is feasible.

**Discussion:**

This kind of study will allow us to estimate correlations between individual intake and household purchases, overcoming the limitations of individual dietary surveys, and enhancing the HBS with information on eating out and intra-familiar distribution of food.

## Background

Data on food intake at the individual level and its statistical distribution in population groups defined by age, gender, or geographic areas are important in planning public health and nutrition programs, such as nutritional enrichment of food during industrial processing, iron supplementation, or regulation of pesticide utilization. However, individual-based surveys (IDS) designed to collect data to estimate individual food consumption in representative population samples are exceedingly expensive, this is why only few developed countries can afford to conduct them on a regular basis.

On the other hand, Household Budget Surveys (HBS) are systematically carried out in many countries, particularly because they are strategic to planning general economic and industrial development programs. Given the national representativeness and the periodicity in data collection, the HBS data are utilized to create an international databank of comparable information, allowing for nutrition monitoring and the estimation of food intake patterns according to demographic and socioeconomic characteristics [1-3]. Although the HBS offers good estimates of trends in eating patterns, the increasing prevalence of eating out may affect interpretations towards the estimation of true intake.

In Brazil, since 1974, the Brazilian Office of Geography and Statistics (*Instituto Brasileiro de Geografia e Estatística *– IBGE) has been conducting HBS on representative samples of the population. Data are collected throughout the year to capture seasonal variations. The protocol covers detailed expenditure patterns, especially regarding food expenses of the household, which includes one week amounts of food acquired for each household, as well as regional prices. Data from the HBS in Brazil have been used to compare trends in household food availability between urban and rural areas, among the different regions of the country, and according to family income strata [4-6].

The most recent survey collected data during 2002–2003 on a sample of about 50,000 households and used information on food purchased, produced, and given as gifts or donations in order to estimate the total household availability of foods, food groups, as well as energy and selected nutrients. Also, the survey included an individual diary to record information on foods eaten out, including types of food, amounts, cost, and the place where the meal was consumed. The quantities originally collected in each household were transformed in food weight, calories and nutrients and divided by family members to allow the *per capita *estimations [6]. *Per capita *food availability in the household has been grouped into 16 major foods or food groups as shown in Table 1.

**Table 1:** Food grouping for Brazilian Budget Surveys and amount of food purchased in kg/per capita/year, 2002–2003, Brazil^1^.

**Food**	**kg/per capita/year**
Cereals	35.5
Beans	12.9
Vegetables (including carrots and beets)	16.0
Potatoes and Roots (cassava, yam, etc)	13.0
Fruits	24.5
Nuts and coconut	1.6
Flour	18.0
Pasta	4.8
Bakery products	20.3
Red meat	16.9
Pork	5.7
Offals (organs)	0.9
Fish	4.6
Poultry	13.9
Eggs	1.7
Milk and milk products	49.9
Sugar & sugar products	23.5
Salt	3.0
Seasonings	3.0
Vegetable oil	8.2
Animal fat (added)	0.4
Alcoholic drinks	5.7
Carbonated drinks	23.9
Hot drinks (coffee, tea, etc.)	3.2
Any processed foods (meat, vegetables, etc)	2.6

^1 ^adapted from Levy-Costa et al, 2005

The experience of gathering data on eating out suggests that it is viable to collect information on overall individual food intake. To reduce costs, the Brazilian government is proposing an IDS to be conducted along with the HBS which will be carried out in 2008–2009. The IDS data will be used to complement the lack of information on individual food consumption in the HBS; furthermore, statistical models will be applied to individualise the household data. In addition, data on household acquisition can be used to evaluate the effect of misreporting, a common source of bias in individual dietary surveys.

This paper presents the methodological framework of dietary data collection and indicates the directions to combining the data from the two methods.

## Methods

### Design of the Brazilian Individual Dietary Survey

#### Study population

The 2008–2009 Brazilian HBS sample will include 60,000 households, assuming that non-response is around 20%. Thirty-percent of the households will be randomly selected to be surveyed on individual food intake. It is estimated that each household has a mean of 3.5 persons aged above 10 years; therefore, individual dietary intake data is expected to be gathered from about 70,000 individuals.

#### Data collection

In the selected households, all family members will be listed, and those aged ten years or more will be included in the individual dietary intake survey. For all children younger than 10 years of age, a separate set of questions will ascertain the place where their meals and snacks are usually eaten; an adult member, usually the mother, will provide this information. Pregnant and breastfeeding women will fill in the individual records; nevertheless their data will not be taken into account in the estimation of individual intake, because their potentially modified food consumption might introduce biases in the estimates. On the other hand, the number of pregnant and breastfeeding mothers is too small for us to gather enough data or to make specific estimations for this group; however, the information on their food intake will be used to compare individual data with the overall family food budget. Proxy information will be recorded for the elderly, children, and for those who are not able to write.

Data collection procedures will comprise the following steps:

(1) Completion of an open diary of food purchases for the household during a seven day period.

(2) Each household member 10 years-old or older will fill out a small notebook registering all items consumed during two non-consecutive days. Water intake will not be considered in the individual data collection. There will be a question regarding usual sugar intake (sugar or sweetener or both). The records must include:

(a) food items;

(b) amount consumed (referred in standard units of volume measurement – usually grams or milliliters – or in universally used cooking ware and tableware – such as cups, plates, tablespoons, ladles, skimmers, etc, or in commonly used serving portions, such as bunch, slices, etc.);

(c) place of the meal (at home or out of home);

(d) time of intake;

(e) preparation of food for specific items (raw, cooked, baked, grilled, fried, steamed, with tomato sauce, sauté, stewed, etc).

(3) Review of the records: an interviewer will visit the household at least twice during the week of data collection to get the filled notebooks on general household acquisitions and individual food intake data. The interviewers will be trained to record dietary data on a computer database, reviewing the individual notes and probing for items usually not mentioned in this kind of records, such as candies, snacks and beverages. Also, when the record shows more than three hours during the daytime without any food intake, the interviewer will make sure that nothing was eaten during that period.

### Computerized datasets and data entry

The computer software designed to store individual data on food intake provides datasets on food items, prepared dishes, cooking methods, and measurements. The building of the food items and prepared dishes list was based on the records of 2002–2003 HBS, where 5,686 food items were reported. A great part of these items were synonyms, however, or had alternative spellings, or yet contained detailed variations that do not present significant differences in nutritional composition (such as different kinds of banana). The review of these items respected regional nomenclature and the diversity of food intake in Brazil. Hence, the computer-assisted interview for the individual dietary intake survey database includes around 1,200 food items and preparations. Interviewers will be able to include more items, but they will be trained to look for the food item that better describes the food reported by the subjects in the dietary records before adding new items.

The food list also includes the names of popular dishes, such as lasagna, pizza, hamburgers, etc. The intake of these food items will be recorded according to the way by which they are commonly referred (i.e., the name of the dish), instead of registering each ingredient (for example, a lasagna will be listed as such, instead of pasta, tomato sauce, cheese, etc).

Each item entered in the individual database will be linked to portions registered in grams, milliliters, plates, tablespoons, cups, etc. The interviewers will not be able to introduce other measurements in the dataset and they will be trained to find the better option in each situation. To provide the standardization of portions, utensils, and tableware, the interviewers' training will include the identification of the measurements listed in the database, and the field manual will include a set of tableware and cooking-ware photographs.

### Data analysis

The sample will be large enough to capture the variations between individuals (**S_b_**), and the two records will assure the estimation of the variation within individuals (**S_w_**) for food groups, energy and nutrients intake, allowing for the deattenuation of individual data for random error.

Data on individual dietary intake (IDI) and food family budget (FFB) will be stratified by the five regions of the country (North, Northeast, Southeast, South, and Central West), and by rural or urban (the latter being subdivided into metropolitan and non-metropolitan). We are anticipating large differences among the five regions of the country as well as among rural, urban metropolitan and urban non-metropolitan areas within each region. Hence, analysis will be accordingly stratified, and for these 15 stratums, statistical models will be constructed assuming the following premises:

1- FFB and IDI are linearly associated (although food intake and household availability may not be linearly associated if there is a significant prevalence of eating out. This assumption will be tested).

2- Households without children younger than 10 years of age yield a good estimation of weekly total intake.

Estimation for the weekly individual food intake (WIFI) as follows:

$$\left[\left(\frac{\text{record \# 1 }+\text{ record \#2}}{\text{2}}\right)*7\right]*\text{de}-\text{attenuation factor}$$

Regression of the sum of all WIFI on FFB for a specific food or nutrient will give the correction factors for FFB data for those items purchased on a weekly basis, such as bread. Therefore, firstly the agreement between WIFI and FFB will be tested and for those foods with correlation greater than 0.80, weekly data will be analyzed. Foods with low correlations indicate that FFB data collected on a weekly basis will not correspond to the household availability, meaning that those foods such as sugar, oil, rice and beans are acquired on a monthly basis. Therefore, for these items showing lower correlations, WIFI will be regressed on the mean of all FFB of those primary sampling unit (about 15 households) of similar income per capita surveyed on a monthly basis. For all food groups, the mean acquisition will be adjusted by family size. These assumptions will be tested comparing household acquisitions and the sum of all WIFI. Models will also take into account all variables that are highly associated with intake, such as household composition, schooling of the head/mother, and income.

Daily energy and nutrients intake will be estimated by transforming the WIFI into calories and nutrients using food composition tables, preferably the Brazilian Food Composition Table [7].

WIFI and FFB data are complementary, and since they are collected through independent tools, they have independent source of errors and can be used for addressing limitations inherent in all data sources.

## Results

### Pilot study

A pilot study was conducted in two states: Santa Catarina, in the South of the country, where 19 households were interviewed, and in Alagoas, in the Northeast, where 27 households were investigated. In these 46 households, a total of 142 individuals contributed with data on family budget, and a sample of 40 subjects, 20 in each city, filled in the two dietary records.

Data on individual food intake were collected for 21 women and 19 men, aged from 10 to 78 years, which reported the intake of 877 food items and the acquisition of 437 items. As expected, since Alagoas is a poorer state than Santa Catarina, a smaller number of food items were reported in that state compared to the latter (mean for household of 10.6 items; standard deviation (SD) = 4.5 in Alagoas *vs*. 11.7 (SD = 4.2) in Santa Catarina).

### Ethical considerations

All ethical related questions are in conformity with the Brazilian Resolution Number 196/96- on research involving human subjects. For all the census and surveys conducted by the bureau of census there is a specific federal law (law number 5534 from November 14, 1968) which guarantees strict secrecy of information gathered, which can only be used for statistical purposes. For future data processing protocols will be sent to the Committee for Ethics in Research at State University of Rio de Janeiro, which monitors the development of the research projects, through annual reports.

Written informed consent will be obtained from each subject by the research assistants. For illiterate participants, verbal consent will be obtained after a detailed explanation of the research protocol.

## Discussion

The pilot study indicated that combining individual and budget data in a survey is feasible. This kind of study will allow for estimating correlations between individual intake and household purchases, overcoming the limitations of individual dietary surveys, such as underreporting, as well as enhancing the HBS with information on eating out and intra-familiar distribution of food.

## Abbreviations

IDS – Individual-Based Survey; HBS – Household Budget Surveys;IBGE – Brazilian Office of Geography and Statistics (*Instituto Brasileiro de Geografia e Estatística*);

IDI – individual dietary intake; FFB – food family budget; WIFI – weekly individual food intake

## Competing interests

The author(s) declare that they have no competing interests.

## Authors' contributions

RS, RAP, AM and ABPAV conceived the study. RS, RAP and AT drafted the paper. All authors read and approved the final manuscript.

## Pre-publication history

The pre-publication history for this paper can be accessed here:


